# Allomelanin: A Promising Alternative to Polydopamine for Bioapplications

**DOI:** 10.3390/jfb17010040

**Published:** 2026-01-15

**Authors:** Silvia Vicenzi, Agata Pane, Chiara Mattioli, Dario Mordini, Arianna Menichetti, Marco Montalti

**Affiliations:** 1 Department of Chemistry “Giacomo Ciamician”, University of Bologna, Via Selmi 2, 40126 Bologna, Italy; silvia.vicenzi4@unibo.it (S.V.); agata.pane2@unibo.it (A.P.); chiara.mattioli20@unibo.it (C.M.);; 2 Tecnopolo di Rimini, Via Campana 71, 47921 Rimini, Italy

**Keywords:** fungi, PDHN, 1,8-DHN, melanin-like nanoparticles, photothermal, nanomedicine, PTT, antioxidant, ROS, radicals

## Abstract

Allomelanin is a natural class of melanin found mainly in fungi and derived from nitrogen-free precursors such as 1,8-dihydroxynaphthalene (1,8-DHN). Despite its biological relevance, allomelanin remains significantly less explored than other synthetic melanin analogs, particularly compared to polydopamine, a synthetic analog of eumelanin. In this review, we provide a comprehensive overview of current knowledge on allomelanin, summarizing the main methods used to characterize its molecular structure, morphology, and chemical functionalities. We also present its emerging applications, ranging from human health to materials science, highlighting how its optical characteristics, ability to modulate redox processes, and antioxidant properties support its growing technological interest. Finally, we describe the natural presence and biological role of allomelanin, highlighting how knowledge of its biosynthetic processes and functions in nature can guide more effective strategies for the design and optimization of new allomelanin materials.

## 1. Introduction

**Figure 1:**
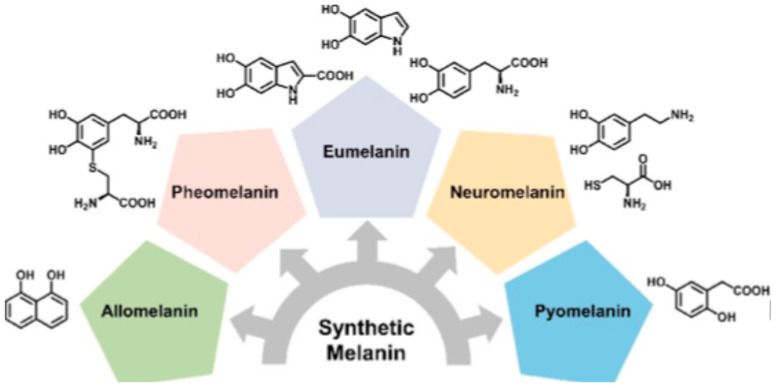
Scheme of the five classes of melanin and the chemical structure of the precursors used in their biosynthesis. Reprinted with permission from ref. [3]. 2021, American Chemical Society.

Melanin is a broad and heterogeneous family of natural polymeric pigments found in living organisms, where it contributes to essential functions such as pigmentation, free-radical scavenging, and protection from radiation and environmental stress [1,2]. Its name derives from the Greek “Melanos,” which means dark or black, reflecting its characteristic coloration. Melanin is formed by the spontaneous oxidative polymerization of different precursors, giving rise to complex and cross-linked structures, stabilized by covalent and non-covalent interactions, that are difficult to fully characterize [3]. For this reason, a simple and effective way to classify the different kinds of melanin is based on the nature of the molecular precursor. Following this strategy, up to now, five different classes of melanin, have been identified: eumelanin, pheomelanin, neuromelanin, pyomelanin, and allomelanin, as schematized in Figure 1.

Between the different kinds of melanin, particular attention was devoted by the scientific community to polydopamine (PDA), the biomimetic, artificial analogous of natural eumelanin [4,5,6,7,8,9,10,11]. PDA found application in a wide variety of fields going from nanomedicine and nanocosmetics [10,12,13,14], to energy conversion and storage [15,16], to environmental remediation [17] and art restoration [18].

**Figure 2:**
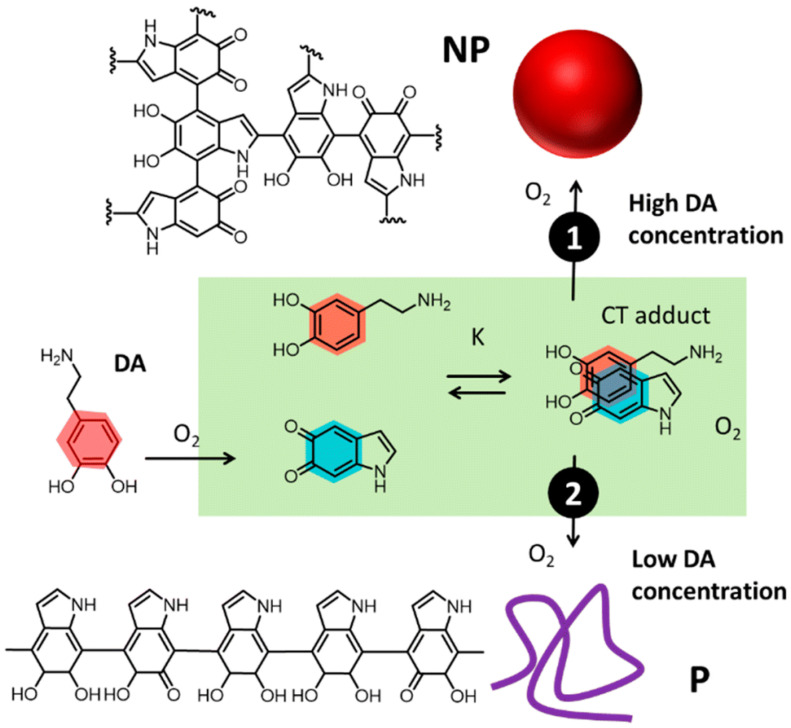
Oxidation and polymerization of dopamine (DA) occurs through two different pathways leading to the simultaneous formation of polydopamine (PDA) in the form of nanoparticles, NP (path 1) and low-density polymers (P). Possible chemical structures are reported. Reprinted from ref. [19].

Use of synthetic PDA as a biomimetic replacement of natural eumelanin was mostly due to the simplicity and versatility of the highly ecologically friendly synthetic processes developed for its preparation [19,20], which presents several advantages, e.g., yields and costs, with respect to the extraction of natural eumelanin [21].The process of formation of PDA from the molecular precursor dopamine (DA) is schematized in Figure 2. The actual chemical composition of PDA is still debated, and possible structures are given in Figure 2. Most relevant features of PDA that make it unique for bio-applications are its biocompatibility, its efficiency as a photothermal agent because of its broad absorption spectrum ant its photostability, and its high activity as a radical scavenger [22,23]. In this paper, we aim to underline that allomelanin shares and, in some cases, surpasses the features of PDA, nevertheless its practical application, even in biology-related fields, is still limited. We hence suggest that biomimetic allomelanin should be considered, in general, as a possible promising replacement for PDA in bio-applications.

**Figure 3:**
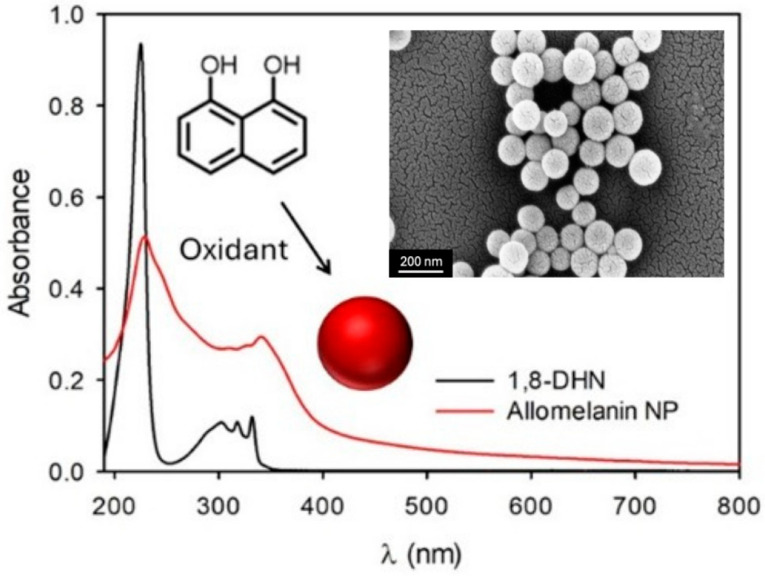
Absorption spectrum of allomelanin NP and of the molecular precursor 1,8-DHN. Scheme of the NP formation is also shown. Inset: SEM image, scale bars 200 nm, of the allomelanin NP. Reprinted from ref. [25].

Allomelanin, mainly found in bacteria and fungi [24] derives from the oxidative polymerization of nitrogen-free precursors as 1,8-dihydroxynaphthalene (1,8-DHN) (PDHN melanin), 1,4,6,7,9,12-hexahydroxyperylene-3,10-quinone (HPQ melanin), and catechol. As schematized in Figure 3, allomelanin can also be prepared artificially from oxidation of 1,8-DHN [25]. In this review, we will primarily focus on PDHN, which is the most extensively studied and biologically relevant form of allomelanin, with a well-defined biosynthetic pathway and abundant natural occurrence in fungi. PDHN is produced through a polyketide-based pathway in fungi, starting from the formation of 1,3,6,8-tetrahydroxynaphthalene catalyzed by a polyketide synthase, and continuing with a series of reduction and dehydration reactions, which give rise to several intermediates, finally obtaining 1,8-DHN. The latter accumulates within the cell wall, where it enhances mechanical robustness, controls permeability, and confers remarkable resistance to environmental stresses, including radiation. These protective capabilities arise from the redox flexibility of the 1,8-DHN repeat units, which can stabilize generated radicals through intramolecular hydrogen bonding, enabling DHN-melanin to act as an efficient electron-transfer buffer. Considering its high efficiency, mimicking the synthesis of PDHN results of great interest in order to obtain synthetic allomelanin materials to be applied to several fields. Synthetic pathways can be chemical or chemoenzymatic. In the chemoenzymatic approach, enzymes such as laccase and horseradish peroxidase (HRP) are employed. Laccases, multicopper oxidases present in plants, fungi, and bacteria, can oxidize a wide range of phenolic compounds and naturally participate in the polymerization of 1,8-DHN.

**Figure 4:**
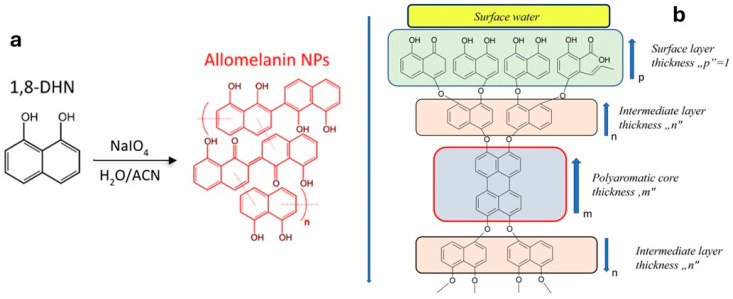
(**a**) Reaction of formation of allomelanin and possible structure according to ref. [25], (**b**) possible chemical structure of allomelanin according to ref. [27]. Reprinted from refs. [25,27].

In this method, 1,8-DHN is dissolved in an acetate buffer and oxidized aerobically in the presence of laccase to form PDHN. Alternatively, HRP catalyzes the oxidation of the monomer in phosphate buffer, with hydrogen peroxide added as the oxidant. The chemical route, by contrast, relies on oxidizing agents such as sodium periodate (NaIO_4_) or potassium permanganate (KMnO_4_), and can be carried out under neutral or mildly acidic conditions. Compared with other synthetic melanins, PDHN assembles from its dimeric or oligomeric building blocks through strong π–π interactions between naphthalene rings and hydrogen bonding involving hydroxyl groups and embedded water molecules. This assembly pathway can be more readily controlled, allowing the formation of more ordered structures that facilitate close contact and improved reactivity with external molecules. Moreover, the morphology of the resulting allomelanin depends on the chosen synthetic methodology. Chemoenzymatic synthesis often leads to poorly defined morphology; instead, with the chemical pathway, well-defined spherical particles can be obtained. Although synthetic melanin is mostly produced as spherical nanoparticles (NPs), as reported by Zhou et al. [26], new routes have been studied for the creation of anisotropic synthetic allomelanins, demonstrating that the building blocks (dimers) of 1,8-DHN can be preorganized into specific shapes, followed by solid-state oxidative polymerization, to access a variety of anisotropic architectures, including sheets and platelets. Synthetic allomelanins retain many of the advantageous properties of their natural counterparts, including strong antioxidant activity, photothermal stability, broad-spectrum absorbance, and the ability to scavenge reactive oxygen species (ROS). As a result, PDHN materials, especially in nanoparticle form, are increasingly recognized as promising candidates for biomedical applications. Their biocompatibility, redox functionality, and structural durability make them attractive for use in radiation protection, oxidative-stress mitigation, photothermal therapy, and drug delivery, positioning PDHN as a rapidly emerging platform in the design of next-generation therapeutic nanomaterials [3]. As for PDA, the chemical structure of allomelanin is not completely known. Possible structures are shown in Figure 4.

## 2. Allomelanin Properties

Although other allomelanin morphologies (such as rods and plates) have also been investigated [26], NPs are the most commonly described type in the literature. These nanoparticles are typically synthesized through two main polymerization routes: enzymatic polymerization and oxidative polymerization, using 1,8-DHN as a precursor. Regardless of the synthesis route, the resulting NPs are characterized using a wide range of complementary techniques that study their optical behavior, size distribution and surface charge, and morphology and ultrastructure, as well as their chemical and molecular composition. In addition, their antioxidant properties, considered a key aspect of their functional behavior, are often studied.

### 2.1. Optical Properties

The optical properties of PDHN NPs are mainly defined by recording their UV-Vis absorption spectrum [22,25,28,29]. This spectrum, regardless of the synthesis method used, shows a characteristic spectral shape, showing a very broad absorption band extending from UV to near IR. Some important differences were observed for allomelanin with respect to PDA [30,31,32,33].

As observed by Petropoulos V. et al. [25], this band has two distinct shoulders, at 470 nm and 580 nm. The shorter wavelength shoulder is believed to be attributed to exciton resonances within the aggregated NPs. This contribution is strongly dependent on the degree of packing and the size of the NPs, and they observed that the shoulder becomes more intense as the size increases and decreases under conditions of disaggregation. The shoulder at 580 nm, on the other hand, is believed to result from exciton transitions in the quinoid oligomers present in the complex structure of the nanoparticles.

In addition to the presence of the two characteristic shoulders, the UV–Vis spectrum of PDHN NPs also shows a marked shift towards longer wavelengths compared to the DHN monomer. In DHN, there are two main absorption bands between 200 and 250 nm and 250–350 nm, attributed to the n → σ*, π → π*, and n → π* transitions [28]. In polymerized NPs, these bands are significantly red shifted, indicating a lowering of the energy of the electronic transitions. Wang et al. [28] investigated the causes of this phenomenon in detail, identifying two main factors. First, the polymerization of DHN into oligomers extends the electronic conjugation and increases the number of chromophores and auxochromes, promoting greater delocalization of π electrons and causing a progressive shift in the bands towards higher wavelengths (red shift). Second, the further self-assembly of oligomers into larger aggregates, stabilized by non-covalent interactions, causes a further shift in the bands towards lower energies, contributing to the typical broad and continuous absorption band observed in PDHN NPs.

In the work of Petropoulos V. et al. [25], PDHN synthesized by oxidative polymerization was also studied using emission spectroscopy. The results show that PDHN has a very low fluorescence yield when excited in the UV, which becomes negligible in the case of excitation in the visible range. These data indicate that PDHN is extremely efficient in dissipating absorbed energy through non-radiative mechanisms, in line with what has been observed for other natural melanins.

### 2.2. Photothermal Properties

The photothermal effect is the release of heat, by dedicated materials, called photothermal agents (PTA), upon absorption of light [34]. Hence, through the photothermal effect, PTA are able to convert radiative energy into heat [35]. The main advantage of this approach to heat generation, with respect to conventional heating, is that it can be achieved very locally (at least micrometric resolution, by focalizing a light beam, and it can be switched ON and OFF very quickly, millisecond time scale or less). This spatial and temporal high resolution makes the photothermal effect an ideal tool for controlled thermal treatments, especially in nanomedicine, and it led to the development of important therapeutic techniques like photothermal therapy (PTT) mostly applied to cancer treatment [36]. PDA, for its biocompatibility, its great photostability, broad absorption extended to the NIR, where biological tissues show the highest transparency, was demonstrated to be a good PTA [37,38,39]. Applications of allomelanin as a PTA is less consolidated than for PDA; nevertheless, as shown in Section 3 of this article, its use for PTT started to be taken into account presenting, as discussed below, important advantages.

### 2.3. Structural and Morphological Characterization

The structural and morphological characterization of PDHN NPs is essential for correlating the physical properties of the material, such as dispersion, stability, and porosity, with its potential biological and technological applications.

Dynamic Light Scattering (DLS) is the most-used technique to determine the hydrodynamic diameter and polydispersity index of PDHN NPs, whether they are obtained by oxidative polymerization or enzymatic polymerization [23,25,28,29]. The literature reports that the final hydrodynamic diameter of nanoparticles, regardless of the synthesis method, falls within the range of approximately 150–300 nm [23,25,29], with a polydispersity index of around 0.1 for oxidative synthesis [23,25,29] and 0.36 for enzymatic synthesis [23]. DLS measurements also allow the kinetics of NPs formation and growth to be monitored and highlight the dependence between the precursor concentration and the final size of the NPs, as demonstrated in the work of Wang et al. [28].

Another technique used to evaluate colloidal stability and surface charge is the measurement of the zeta potential [22,40]. PDHN NPs generally show a negative zeta potential, between −27 mV and −33 mV, indicative of high colloidal stability in aqueous solution [23].

**Figure 5:**
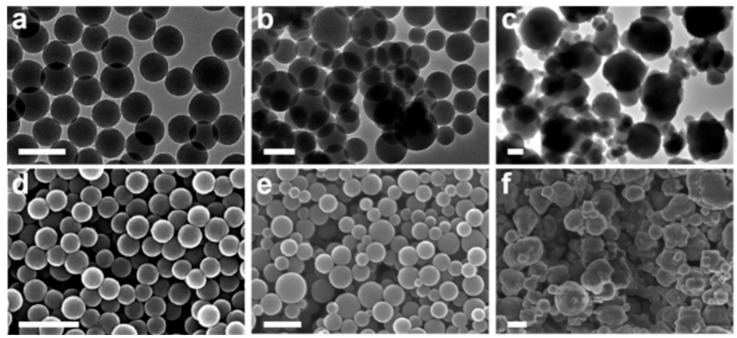
Characterization of the morphology and size of PDHN NPs by TEM (**a**–**c**), scale bars 200 nm, and SEM (**d**–**f**), scale bars 400 nm. Electron micrographs of (**a**,**d**) PDHN NPs synthesized with NaIO_4_, (**b**,**e**) PDHN NPs synthesized with KMnO_4_, and (**c**,**f**) PDHN NPs synthesized with laccase. Reprinted with permission from ref. [23]. 2019 American Chemical Society.

For further confirmation of the morphology and size of PDHN NPs, transmission electron microscopy (TEM) and scanning electron microscopy (SEM) are the techniques most frequently reported in the literature [23,25,28,29,40], as they allow direct visualization of the shape and size of the synthesized NPs, as shown in Figure 5. These techniques confirm that the morphology of PDHN NPs can vary from monodisperse spheres to other structures, such as sheets [26]. An additional technique used by McCallum et al. [22] is atomic force microscopy (AFM), which is used to obtain high-resolution images of the particle surface, complementing the morphological information provided by SEM. SEM is also used to study the behavior of nanoparticles, in particular, self-assembly processes [40] and their deposition on composite films [28].

In addition to the most common microscopic techniques, such as TEM and SEM, McCallum et al. [22] investigated the morphological characterization of PDHN NPs using advanced electron microscopy techniques, with the aim of verifying the hypothesis that PDHN NPs have intrinsic microporosity, attributable to the inefficient packing of oligomers derived from the oxidation of 1,8-DHN. In particular, the combined use of bright-field STEM (BF-STEM) and high-angle annular dark-field STEM (HAADF-STEM) allowed the visualization of variations in electron density within the particles, highlighting less dense or partially emptied regions attributable to the presence of a porous internal structure.

### 2.4. Chemical Characterization

The molecular and chemical characterization of PDHN NPs is generally based on a set of complementary spectroscopic and chromatographic techniques, used to clarify both the functional groups present in the NPs and the oligomerization processes that the precursor undergoes during polymerization.

One of the most widely used techniques for studying the main functional groups present in PDHN is Fourier Transform Infrared (FTIR) Spectroscopy [23,25,28]. As can be seen from the study by Petropoulos V. et al. [25], the oxidative polymerization process causes a broadening of the bands in the 3200–3400 cm^−1^ range associated with the stretching of the –OH and –CH bonds on the naphthalene ring, due to the increase in geometric and chemical disorder in the structure. There is also a suppression of the aromatic C-H bending peak at 753 cm^−1^, indicative of intermolecular coupling of the naphthalene rings in the formation of PDHN NPs.

In the work of Wang C. et al. [28], X-ray Photoelectron Spectroscopy (XPS) and Electrospray Ionization Mass Spectrometry (ESI-MS) were also used to investigate the formation mechanism of PDHN NPs. XPS analysis was used to track the evolution of the surface elemental composition, showing a higher percentage of C=O groups in PDHN NPs than in the monomer. This suggested that the oxidation of the 1,8-DHN monomer led to the conversion of some of the phenolic groups into quinone structures. ESI-MS, on the other hand, allowed the detection of monomers, dimers, and intermediate oligomers, providing information on the early stages of oxidation and aggregation leading to the formation of NPs.

Zhou X. et al. [23] investigated the formation of oligomers using techniques such as HPLC, ESI-MS, LC-MS, and MALDI-TOF, which allowed the separation and identification of the different oligomeric species constituting PDHN NPs. Solid-state nuclear magnetic resonance (ssNMR) analysis was also performed to investigate the molecular architecture of the material. This technique provided information on the chemical environment of the carbon nuclei, confirming the presence of aromatic structures and oligomeric units consistent with the oxidative coupling and self-assembly mechanisms typical of PDHN.

The literature also reports examples of the use of techniques such as FTIR and XPS to analyze post-synthetic modifications of PDHN NPs, as described in the work of Zhou X. et al. [40].

### 2.5. Antioxidant and Radical Scavenging Properties

Antioxidant activity and radical scavenging activity consider different processes [41], nevertheless a correlation between these two kinds of action is often accepted [42,43]. However, radical scavenging activity of a substance can be easily estimated using spectrophotometric tests based, for example, on the degradation of colored radical species like the 2,2-Diphenyl-1-picrylhydrazyl one (DPPH) [44]. These tests can be performed fast and easily and they do not require a dedicated instrumentation as for the determination of the antioxidant activity [45].

The antioxidant activity of PDHN is generally attributed to its remarkable ability to scavenge free radicals, as suggested by Zhou X. et al. [23], who showed that PDHN NPs exhibit the existence of free radicals, identified by EPR spectroscopy, and possess marked antioxidant activity as assessed by the 2,2-diphenyl-1-(2,4,6-trinitrophenyl) hydrazine (DPPH) assay. The results indicate that the free radical scavenging capacity of PDHN NPs is comparable to that of ascorbic acid, a well-known antioxidant and it is superior to PDA.

This result suggests that the use of allomelanin as a possible replacement for PDA should be considered for all the applications that take advantage of an effective antioxidant activity.

Petropoulos V. et al. [25] studied the increase in antioxidant activity of PDHN NPs induced by exposure to light, observing that irradiation increases the content of radicals and promotes the formation of semiquinone-type radicals. Using the DPPH assay and comparing measurements taken in the presence and absence of light (specifically ambient sunlight and 400 nm violet LED light), an increase in antioxidant activity was observed. The results therefore indicate that PDHN NPs not only effectively absorb UV-NIR light but can also exploit light energy to enhance their antioxidant capacity.

A more detailed study by Mavridi-Printezi A. et al. [29] analyzed the antioxidant activity of PDHN-NPs compared to polydopamine nanoparticles (PDA NPs), experimentally and theoretically evaluating their ability to neutralize alkylperoxy (ROO•) and hydroperoxy (HOO•) radicals, which are involved in the propagation of auto-oxidation. In water, PDHN NPs exhibit antioxidant activity that is absent in PDA NPs, attributed to residual phenolic groups. In acetonitrile, PDHN NPs are also more effective than PDA NPs in neutralizing ROO• and HOO•, thanks to catalytic cross-termination activity. Quantum mechanical calculations indicate that this superior reactivity derives from the presence of extended quinones with high affinity for the hydrogen atom, supported by a network of hydrogen bonds, thus explaining the rapid reaction with HOO• radicals.

## 3. Applications

### 3.1. Human Health

PDHN NPs have recently gained attention as biocompatible nanomaterials with strong antioxidant, photothermal, and free radical-scavenging properties. Their unique stability, ROS-neutralizing capacity, and tunable surface chemistry make them promising candidates for a wide range of medical applications, including cancer therapy, tissue protection, and treatment of oxidative stress-related diseases. Examples discussed in this section are summarized in Table 1

Regarding cancer treatment, PDHN has been involved in the treatment of Glioblastoma (GBM), one of the most aggressive tumors of the central nervous system, by Sun and co-workers [46]. Classical treatments, such as chemotherapy and radiotherapy, can lead to diverse side effects, and they are limited by the presence of the blood–brain barrier (BBB), which needs to be crossed by specific drugs to exert their power in GBM tissues. The authors relied on modern therapeutic approaches involving photothermal therapy (PTT), in which photothermal conversion agents (PCAs) convert near-infrared (NIR) light into localized heat, killing tumor cells while sparing healthy tissues. PCAs should be able to overcome the BBB and accumulate in GBM tissue, beyond the need to have high biocompatibility. To increase their permeability, the authors developed cancer cell membranes (CCMs) with which to coat nanocarriers, obtaining high selectivity and permeability to treat tumors in vivo. They further proposed advancement in GBM treatment, exploiting the synergistic activity of PTT and immunotherapy, activating the patient’s immune system to enhance tumor cell death. In particular, they relied on immune checkpoint blockade (ICB) therapy, a form of cancer immunotherapy that boosts the body’s ability to fight tumors by blocking inhibitory signals that normally shut down T-cell activity. Tumors often overexpress molecules such as PD-L1 that bind to immune checkpoints like PD-1 on T cells, weakening the immune response and allowing cancer to escape detection. ICB therapy can block these interactions by relying on antibodies, releasing the brakes on the immune system. The authors developed a biomimetic nanoplatform (PDHN@CLP@CCM) for GBM-targeted PTT and ICB synergistic therapy by loading CLP002, an immune checkpoint inhibitor, into the PDHN NPs and then coating them with CCMs. PDHN NPs served both as photothermal agents for PTT and as nanocarriers for CLP002 delivery. Coating the PDHN@CLP nanoparticles with CCM greatly improved their ability to cross the BBB and selectively target GBM, as confirmed through both in vitro and in vivo tests. Experimental results showed that PDHN NPs provide strong photothermal tumor ablation, triggering apoptosis and immunogenic cell death, while the CLP002 peptide effectively blocks PD-L1. When combined, PTT and ICB markedly boost T-cell activation and alleviate the immunosuppressive tumor microenvironment, leading to potent inhibition of orthotopic GBM. With excellent biocompatibility, efficient BBB penetration, and robust immune activation, this nanoplatform showed strong promise for treating orthotopic GBM.

**Figure 6:**
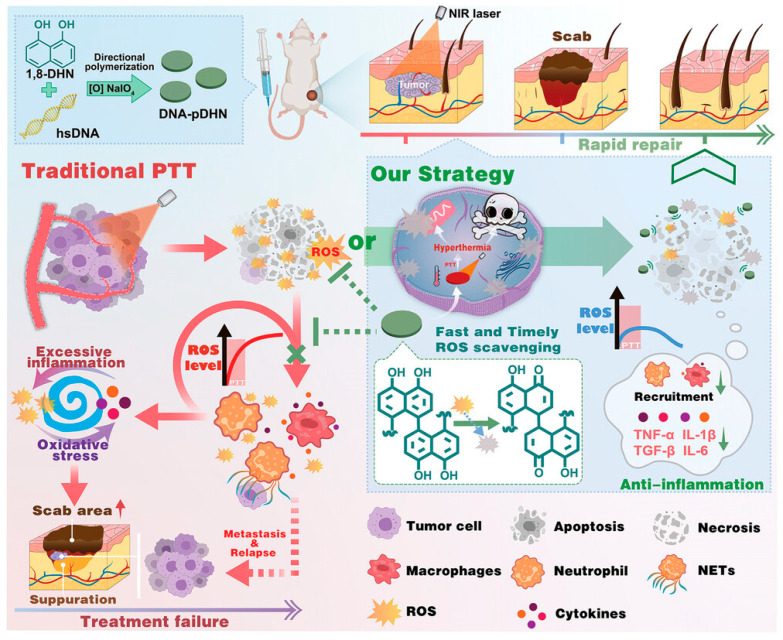
The preparation of DNA-pDHN nanodisks and the designed strategy to accelerate burn tissue repair and inhibit tumor metastasis/relapse after PTT by fast scavenging of heat stress-induced ROS. Reprinted from ref. [47].

PTT has emerged as a powerful strategy for treating tumors. However, the intense heat required for tumor ablation inevitably triggers strong inflammation, characterized by excessive production of inflammatory cytokines and ROS. This harmful inflammatory response can enlarge tissue damage, delay wound repair, and create an immunosuppressive microenvironment that promotes tumor recurrence and metastasis. Heat-induced ROS, in particular, play a central role in establishing this destructive feedback loop. Even brief NIR exposure can activate immune cells, accelerate ROS formation, and disrupt tissue redox balance, causing runaway inflammation during and after PTT. Most current PTT systems do not actively control this early oxidative burst; anti-inflammatory drugs introduced afterward respond too slowly or only passively. Thus, Wang et al. fulfilled the need for PTT agents capable of rapidly scavenging ROS during irradiation, preventing the onset of pathological inflammation [47]. They relied on melanin-based nanomaterials, which have been explored for PTT because of their photothermal efficiency and inherent antioxidant properties. Still, previous PDHN NPs were primarily spherical, making many internal phenol groups inaccessible. To address this, the authors proposed a strategy using DNA to direct the assembly of PDHN oligomers into nanodisks with ordered stacking, maximizing exposure of reactive phenolic groups while maintaining strong photothermal performance. The study successfully developed DNA-guided PDHN nanodisks (DNA-pDHN) as a novel photothermal agent capable of simultaneously delivering high photothermal efficiency and ultrafast ROS scavenging (Figure 6). Their ordered nanoscale structure exposes a large number of phenolic groups, enabling exceptionally strong and rapid antioxidant activity. Moreover, PTT triggers partial structural disintegration of the nanodisks, further amplifying their ROS-scavenging effect. In vitro and in vivo experiments demonstrated that these nanodisks achieved effective tumor ablation while preventing the harmful inflammatory cascade typically associated with PTT. By rapidly reducing ROS levels, the nanodisks disrupted the positive-feedback loop of oxidative stress and inflammation. This controlled inflammatory environment led to accelerated tissue repair, enhanced M2 macrophage polarization, suppression of neutrophil extracellular traps (NETs), and strong recruitment of CD4^+^ and CD8^+^ T cells. As a result, the treatment not only promoted wound healing but also significantly reduced tumor metastasis and relapse after PTT. Overall, the rationally engineered, redox-active nanostructures, such as DNA-pDHN nanodisks, represent a powerful strategy for improving PTT outcomes by integrating efficient photothermal effects with dynamic ROS control.

As is well known, diabetes mellitus is a widely spread chronic disease that causes damage to the vascular and neurological systems of patients. In particular, diabetic wounds are highly susceptible to infection due to the presence of ROS and impaired synthesis of nitric oxide (NO), leading to a restriction in nutrient and oxygen transport, which can cause harmful consequences, such as amputation of the infected portion. He et al. developed a dynamic crosslinked hyaluronic acid (HA) hydrogel dressing (Gel-HAB) loaded with PDHN, N′-dis-sec-butyl-N, N′-dinitroso-1, 4-phenylenediamine nanoparticles (PDHN-BNN6) for healing diabetic wounds [48]. Hyaluronic acid is involved as a biological signaling molecule that can promote cell migration and proliferation, thus accelerating tissue regeneration. In addition, the presence of antibacterial and antioxidant agents, as PDHN, is needed to kill bacteria and impede biofilm formation and to quench the presence of ROS, processes that contribute to the inhibition of inflammation. PDHN is used as a photothermal antibacterial therapy agent, exerting its properties when heated up to 60 °C. However, the high temperature could damage the surrounding healthy tissues. To address this problem, a gas therapy based on NO was involved in order to increase the antibacterial power of the system even at lower temperatures (<50 °C). NO is essential because it acts as a potent broad-spectrum antibacterial agent that synergizes with PTT to achieve effective sterilization at a safer, mild temperature (<50°), preventing the high-temperature damage caused by PTT alone.

To create a potent diabetic wound dressing, the researchers first synthesized PDHN-BNN6 NPs by immobilizing the NO donor (BNN6) onto PDHN NPs via π-π stacking, yielding a material with antioxidant, photothermal, and NO-releasing capabilities. These NPs were then incorporated into a mixture of modified Hyaluronic Acid (HA-ADH and OHA) to form an injectable, self-healing HA hydrogel (Gel-HAB) through dynamic acylhydrazone cross-linking. The resulting Gel-HAB dressing effectively scavenged ROS, released NO under NIR light to enable mild photothermal antibacterial therapy, and ultimately demonstrated significant improvements in diabetic wounds by reducing oxidative stress, controlling infection, promoting angiogenesis, and accelerating healing.

A similar approach to treat diabetic wounds was assessed by Bi et al. [49]. As the previously cited research, they developed an injectable hydrogel dressing (PQCS/OD/PDHN@Cur). The goal of this system was to modulate the neuroimmune microenvironment and enhance diabetic wound healing. Schwann cells (SC), which form the myelin sheath around peripheral nerves, can be activated by certain neuropeptides to improve skin wound healing through TGF-β3. However, in diabetic patients, the number and function of peripheral nerves and SCs are severely reduced. Since SC are essential for nerve survival and regeneration, the aim of the authors was to restore their activity in diabetic wounds to speed healing. Macrophages also play a key role: M1 macrophages promote inflammation, while M2 macrophages help repair nerves by supporting SC growth and migration. In diabetic wounds, constant oxidative stress disrupts the balance between M1 and M2 macrophages, causing a major drop in M2 levels and weakening SC-mediated repair. Therefore, treatments that reduce oxidative stress and restore the balance between macrophages and Schwann cells could significantly improve neuropathy and enhance healing in diabetic wounds. For this reason, Curcumin (Cur), which possesses strong antioxidant properties, was involved in the system to scavenge ROS and to enhance proliferation and myelinization of SC. In order to achieve controlled release of Cur, PTT was involved, further enhanced by loading Cur into artificial PDHN NPs, which show excellent photothermal properties. In this system, polyaniline-grafted quaternized chitosan (PQCS) was involved because it supplies the hydrogel with electrical conductivity, antibacterial activity, and abundant amine groups that participate in crosslinking. Oxidized dextran (OD) further provides the complementary aldehyde groups needed for the Schiff base reaction with PQCS, enabling the formation of an injectable and self-healing hydrogel network. Together, PQCS and OD create a conductive, biocompatible matrix that supports ROS scavenging, controlled drug release, and enhanced healing of diabetic wounds. Results obtained on the peripheral nerve injury model and infected diabetic wounds showed significant promotion of neurogenesis, reduction in ROS, and restored balance between M1 and M2 macrophages, enhancing the recovery of diabetic wounds.

Thanks to its free-radical scavenging ability, PDHN was employed to mitigate radiation-induced injury at the intestinal mucosa of the human body by Zhang and colleagues [50]. PDHN can be synthesized or accumulated in microbes such as fungi. Melanized fungi have been proven to survive extreme conditions, including elevated radiation levels. Considering the gut microbiome, which plays a crucial role in health and diseases, a fundamental aspect to consider when employing melanized bacteria in the human body is their biocompatibility. In their research, Zhang and colleagues evaluated the effects of soluble melanin and melanized bacteria on the gut microbiome of mice in order to evaluate their safety. They administered mice with soluble PDHN and with melanized bacteria (*E. coli* Nissle, a probiotic bacterial strain with unique biological and therapeutic properties) and observed changes in body weight and intestinal physiology of the treated mice. Overall, this study provides the first in vivo evidence supporting the safety and biological behavior of both soluble PDHN and melanized *E. coli* Nissle as potential radiation-injury mitigators. Melanized *E. coli* Nissle demonstrated rapid and sustained colonization of the colon within 3 h and up to 24 h post-administration, while soluble PDHN showed no adverse effects on body weight or gut microbiome components. Moreover, neither agent induced intestinal inflammation. Although the work was limited to early effects and a partial microbiome assessment, the findings establish a strong safety foundation for these melanin-based interventions and justify future long-term studies aimed at mitigating radiation injury and restoring microbiome health.

PDHN NPs have been demonstrated to be promising candidates in antioxidant therapies. They have been involved by Chen et al. to treat sensorineural hearing loss (SNHL), an auditory pathology attributed to oxidative stress-related mechanisms, including the cytotoxic effects of specific drugs as cisplatin [51]. Cochlea has high metabolic demands, and it is very sensitive to ROS, which increases after noise exposure or cisplatin treatment and causes lipid damage, cell death, poor blood flow, and inflammation. The authors developed an innovative antioxidant strategy based on PDHN NPs to confer auditory protection against cisplatin-induced and noise-induced cochlear damage. Compared with dexamethasone, a standard clinical treatment, the PDHN NPs demonstrated strong antioxidant effects and reduced inflammation in both cell models and animal models, highlighting them as promising new nanomedicine for treating SNHL.

Lastly, PDHN NPs have been employed in electrochemical sensors for the detection of target compounds by Chandran and colleagues [52]. They developed a novel composite (AM@UIO-66-NH2) for the detection of dopamine in human serum. Dopamine, present in the extracellular fluid of brain nuclei, is a neurotransmitter that plays a crucial role in brain functions and behavior. However, high levels of dopamine can lead to several diseases, such as Alzheimer’s disease and Parkinson’s syndrome. Dopamine is commonly detected by different analytical techniques, but they are complex and require long operation times and expensive equipment, limiting their applicability. To address these limitations, the authors approached a new methodology, relying on electrochemical sensors, which offer low-cost, simple, highly selective, and sensitive features to enhance dopamine’s detection. They employed unique nanomaterials, Metal–Organic Frameworks (MOF), to enhance signal amplification. They are crystalline, porous materials composed of metal ions bound by organic ligands via coordination bonds. Among them, UIO-66-NH_2_, an amino-functionalized Zr-based MOF, was chosen for electrochemical sensing because its amine groups help bind analytes and immobilize biological ligands like aptamers. However, MOFs generally suffer from low electrical conductivity, which limits their sensing performance. To overcome this issue, polymers, PDHN in this case, are often used to modify MOF composites and improve conductivity. PDHN offers useful properties such as strong radical-scavenging ability, adhesiveness, biocompatibility, and radiation protection. The developed sensor was characterized via spectroscopic and analytical techniques in order to evaluate its morphology, composition, functional groups, crystalline phases, thermal stability, and electrochemical properties. Experiments were performed in real samples using human serum, achieving a low limit of detection and a linear dopamine detection range. Moreover, the sensor demonstrated excellent selectivity and sensitivity to successfully detect and quantify dopamine in real samples.

### 3.2. Material Development

Although the application of PDHN in human health is highly significant, it is not the only field in which this biomolecule is valuable. The following paragraph highlights its importance in materials science, particularly for its roles in sustainable water remediation, as a photothermal synergistic catalytic agent and adsorbance boosting agent, for enhancing mechanical properties, providing multi-radiation resistance, and as a porous coating or molecular-level reinforcing agent for selective separation. One compelling area where PDHN serves as an invaluable material component is in addressing urgent environmental challenges, such as water contamination.

#### 3.2.1. Sustainable Water Remediation Processes

**Figure 7:**
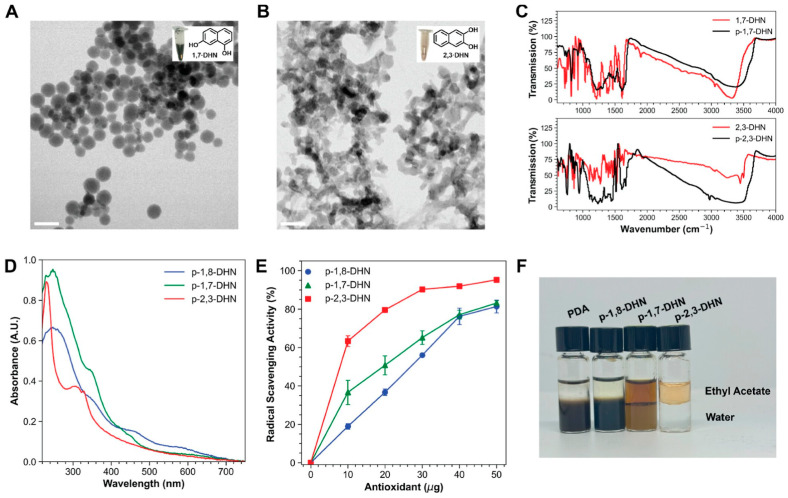
Synthesis and characterization of allomelanin-inspired nanomaterials. TEM images of (**A**) 1,7-DHN-NPs and (**B**) 2,3-DHN-NPs. Scale bar = 200 nm. (**C**) FTIR spectra of p-1,7-DHN/1,7-DHN (**top**) and p-2,3-DHN/2,3-DHN (**bottom**) (**D**) UV/vis absorption spectra. (**E**) DPPH radical scavenging activity (**F**) Solvent partitioning of melanin nanomaterials into ethyl acetate and water. Reprinted with permission from ref. [57]. 2025 American Chemical Society.

Industrialization leads to significant discharge of organic pollutants, primarily dyes and phenolic compounds, into environmental waters, leading to adverse health effects for humans and other life forms. The resulting high levels of water pollution and the inadequate provision of safe drinking water have stimulated research to develop increasingly effective water cleanup methods. Two different research groups approach this problem by developing PDHN functionalized systems, tested by removing methylene blue from water. Lino et al. in 2023 designed and fabricated an innovative water filtering device based on a nylon membrane coated with a synthetic melanin polymer inspired by fungal allomelanins and generated via the oxidative polymerization of 1,8-DHN [53]. They demonstrated for the first time the ability of 1,8-DHN derivatives materials to efficiently sequestrate the azo dye methylene blue both in batch experiments, when used as bulk nanoparticles, and in in-flow experiments, when used as coating of nylon membranes (Figure 7). Building on these advances in melanin-based water purification, another research group, Liu et al., has explored the catalytic and photothermal capabilities of natural melanin and synthetic PDHN supports [54]. In this study, silver nanoparticles were deposited onto sepia eumelanin (SE) and artificial PDHN through an in situ reduction strategy, yielding Ag/SE and Ag/PDHN NPs composites. The process by which these nanocomposites work involves a mechanism known as photothermal synergistic catalysis, which accelerates the catalytic reduction in organic pollutants methylene blue and 4-nitrophenol. Comparative tests confirmed that nitrogen and oxygen rich functional groups on the SE surface were essential for uniformly loading the silver nanoparticles, leading to much greater performance than the nitrogen-free control catalyst, Ag/PDHN NPs. The unique nanostructure of SE enabled efficient silver loading and provided high light absorptivity, effective charge separation, and superior photothermal conversion, collectively accelerating the degradation of organic pollutants. These findings highlight the potential of melanin-based, photothermally active catalytic systems as cost-effective, sustainable platforms for advanced wastewater treatment.

Beyond water purification and catalytic applications, the structural integrity and inherent porosity of PDHN also render them excellent materials for precise molecular separation.

#### 3.2.2. Separation of Hexane Isomers

The large range of applicability of melanin give Su et al. an optimal alternative to traditional polymer-coating MOFs which normally maximize the polymer properties at the surface but results in the dramatic loss of MOF porosity due to blockage by the nonporous polymeric coating [55]. They utilized microporous synthetic PDHN as a porous coating on the zirconium-based MOF (Zr-MOF) UiO-66 via an in situ surface constrained oxidative polymerization and demonstrated through nitrogen sorption isotherms that it does not inhibit access to the porous MOF core. The core shell structure was confirmed by TEM, EDS, and PXRD analyses. By varying the feed ratio of monomer and MOFs they were able to tune the PDHN coating thickness on UiO-66 affecting the hierarchically porous structures of these AM@UiO-66 composites engender excellent hexane isomer separation selectivity and storage capacity.

Moving beyond separation applications, the intrinsic PDHN photochemical properties, as its strong UV absorption and potent ROS scavenging, makes it particularly well-suited for improving the durability and resilience of polymeric materials against environmental damage.

#### 3.2.3. Multi-Radiation Resistance Application

The need to protect polymeric materials and organisms from radiation damage, particularly UV and ionizing radiation, has driven the search for innovative remedies. They found in PDHN a promising solution due to its ability to absorb UV radiation and perform radical scavenging. This nitrogen-free variant of melanin can be found in fungi resistant to extreme conditions, such as those found near Chernobyl. These nanomaterials, which demonstrate superior UV absorption capacity compared to polydopamine (PDA) and potent radical scavenging properties, have been incorporated into polymer matrices such as polyurethane (PU), fluorinated polyimide (FPI), and polyvinyl alcohol (PVA), by different research groups. Wang et al. initially focus on a green enzymatic polymerization strategy to efficiently and eco-friendly synthesize PDHN-based artificial NPs starting from 1,8-DHN [28]. Then, the PDHN NPs were incorporated with PVA, creating a UV shielding film, whose potential was tested by employing it to design a sunroom for safeguarding plants. The system showed a significant advantage in plant height and outperformed in the germination index and vigor index without compromising the light intensity required for healthy plant growth. Furthermore, they prove that these wide-bandgap PDHN NPs efficiently generate hydroxyl radicals under UV irradiation, resulting in excellent in vivo and in vitro antibacterial properties that expand their potential applications.

Differing from the previous study Lu et al. focus mostly on reinforcing mechanical film properties other than just focusing on UV-shielding [56]. Instead of PVA as film matrix they used FPI then filled with PDHN NPs. The addition of PDHN NPs enhances the interaction and entanglement between the FPI molecules, creating a hydrogen-bonded network structure. This incorporation results in elevated tensile strength, fracture elongation, and UV shielding, due to the NPs dual action of altering molecular packing, which affects stress transfer, and providing UV absorption and free radical trapping capabilities. The excellent UV shielding was demonstrated by UV-Vis spectroscopy, curcumin photodegradation analysis, and repeated running tests.

Taking this a step further, Irie et al. extended the topic to ionizing radiation resistance by using DHN isomers 1,7-DHN and 2,3-DHN [57]. The nanomaterials obtained by the oxidatively polymerizing of those precursors have been dispersed in PU elastomer as matrix and they obtained a film with tunable mechanical properties and optical transparency. At a low loading, the PDHN-inspired nanomaterials provide excellent UVB protection to the PU matrix, preserving its mechanical properties and surface chemistry, and suppressing deep crack formation. These PU composites exhibit enhanced tensile mechanical properties and demonstrate robust multi-radiation protection, maintaining their performance after exposure to both UVB and gamma irradiation. This indicates the great potential of P-2,3-DHN and P-1,7-DHN-based nanomaterials for generalized radiation protection applications.

These works highlight the versatility of PDHN-inspired nanomaterials across multiple polymer systems, where their unique capacity for UV absorption, free-radical scavenging, and molecular-level reinforcement consistently translates into enhanced mechanical stability and radiation resilience.

## 4. Allomelanin in Nature

In the previous sections, we established that PDHN is an abundant, versatile, and underexplored pigment with significant biological and industrial relevance. Across fungi, bacteria, plants, and even edible mushrooms, its biosynthesis is governed by diverse metabolic pathways and environmental signals, yet it consistently contributes to UV protection, antioxidant activity, structural coloration, and ecological resilience. The growing demand for sustainable, low-cost melanin sources has fueled studies into new biological producers and environmental factors influencing melanin accumulation. Collectively, recent studies, ranging from fungal extraction and microbial synthesis to light-regulated mushroom pigmentation, demonstrate the remarkable biochemical capability and broad applicability of PDHN. Examples discussed in this section are summarized in Table 2.

**Figure 8:**
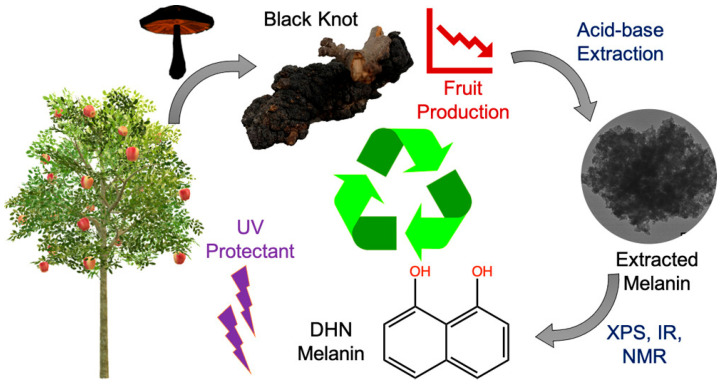
Schematic representation of DHN melanin acid-base extraction from Black Knot fungi. Reprinted from ref. [58].

The potential of fungi, as PDHN producers, was explored by two different research groups. While Singla et al. relied on the pathogenic black knot fungus (*Apiosporina morbosa*) as a source of PDHN, Qui et al. used a highly sought-after edible mushroom (*Morchella sextelata*) for studying the effects of blue light on cap pigmentation. Singla et al. demonstrate that melanin can be extracted from the black knot fungus with a yield of ∼10% using the acid−base extraction method, as schematized in Figure 8 [58]. The extracted melanin was characterized using XPS, IR, and ssNMR spectroscopy. These analyses revealed that the melanin obtained is indeed PDHN with a broadband UV absorption and an irregular morphology. Researchers proved that black knots’ low cost, wide availability, and invasive fungal origin make them a promising, cheap green source of PDHN for UV-absorbing and antioxidant uses. In contrast, Qui et al. provided the first evidence that blue light significantly enhances cap pigmentation in *Morchella sextelata* by promoting melanin synthesis. Increasing blue-light intensity led to greater melanization, with microscopy showing melanin localized within mycelial structures. Spectroscopic and chemical analyses revealed that both brown and black caps contain three types of melanin: eumelanin, pheomelanin, and allomelanin, but blue light stimulates the production of just the first two. Their findings reveal a blue-light photoinduction mechanism regulating melanization and offer new insights for improving *M. sextelata* cultivation and understanding light-mediated pigmentation in mushrooms [59].

Beyond fungal systems, another promising strategy for accessing PDHN relies on microbial synthesis. Ahn et al. in their study proposed a synthesis of PDHN starting from wild-type Streptomyces glaucescens and recombinant *Escherichia coli* BL21(DE3) strains, from caffeic acid, L-tyrosine, and L-lysine. The melanin yield from S. glaucescens was substantially higher than that of *E. coli*, though still lower than the yields achieved through fungal extraction. The extracted allomelanin showed strong antioxidant activity and was applied in cotton dyeing, where laccase treatment significantly improved dye speed by enhancing melanin oxidation and polymerization. It is suggested that the laccase enzyme treatment might induce radical formation in the melanin structure, leading to the tighter binding of melanin to the cellulose that constitutes cotton fibers. Moreover, the researchers investigated the supply of C5-diamine, a crosslinking reagent, in the synthesis of caffeic acid-based melanin, aiming for higher production and novel functionalities. The step further researchers take was engineering *E. coli* strains by introducing combinations of specific enzymes to facilitate the microbial production of melanin components, particularly caffeic acid and the crosslinking C5-diamine, using simple starting amino acids (L-tyrosine and L-lysine). Overall, the study demonstrates the feasibility of microbial PDHN production and highlights its potential as a functional, eco-friendly biomaterial for applications such as textile dyeing and future melanin-based polymers [60].

Whether extracted from fungi or produced via engineered microbes, PDHN exhibits valuable antioxidant properties, UV protection, and functional applicability in industrial processes such as textile dyeing. Advances in understanding its biosynthesis, environmental regulation, and enzymatic modifications open new avenues for sustainable production and novel material applications.

## 5. Critical Evaluation, Perspective, and Proposed Future Directions

The main purpose of this review article is to demonstrate that allomelanin may represent a valuable alternative to other melanin-related materials, and, in particular to PDA, for important bio-applications. The reason for this is that during the last decades PDA was used, in virtue of its optical, electronic, and chemical properties, as a unique component for the design of innovative nanodevices and materials not only in the fields of biology and medicine but also for energy processing and for the preservation of the environment and of artworks. On the other hand, several studies demonstrated that allomelanin can present even better properties than PDA, especially when antioxidant and radioprotective activity are considered. The synthesis of allomelanin, on the other hand, can be performed in conditions quite similar to PDA, although a stronger oxidant than atmospheric oxygen may be needed. In view of future application. Results discussed in this review paper show that allomelanin can be actually used as a promising alternative to PDA. Nevertheless, some fundamental studies are still missing for this material, hence we suggest scientists to consider the following points.

Investigating the film-forming ability of allomelanin. One of the most appreciated features of PDA is its ability to form thin films on the surface of almost any kind of material, hence, in simple words, its stickiness [61,62,63,64]. Thanks to this property, it is extremely simple to grow a layer of melanin on a surface or on nanoparticles to increase their bio-compatibility or density of functionalization [65,66]. For allomelanin this ability has not been reported and, more in general, no systematic study about the experimental conditions suitable to achieve this behavior (attachment to other materials) was performed. For PDA itself it is well known that different morphology are obtained in different experimental formation conditions (e.g., changing pH or the kind of buffer) going from films, to water soluble polymer to nanoparticles.

Developing biocompatible oxidants for the synthesis of allomelanin. Formation of allomelanin, is, as for other melanin-like materials, a process of oxidative polymerization of a precursor such as, e.g., 1,8-DHN. Some recent studies showed that atmospheric oxygen can be used for allomelanin production [27]. Nevertheless, strong and less biocompatible oxidant, e.g., KMnO_4_, are used for the oxidation of 1,8-DHN. The nature of the oxidant has been reported to affect the properties of the produced allomelanin [22]. A systematic study of the effect of oxidant on allomelanin properties should be performed and the possibility of using biocompatible oxidants for allomelanin synthesis should be investigated more in detail.

Functionalization of allomelanin. Ease of functionalization is a strong advantage of PDA [17]. This is mostly the result of the variety of functional groups present in this material [67,68]. Beside the absence of nitrogen atom, allomelanin is also expected to be very suitable for functionalization. No systematic studies are presently available that consider this issue.

Graphitization of allomelanin. Modification of PDA with laser light has been proposed as a method to increase the strength of the coating without reducing the chemical functionalities [69] by graphitization. Possibility of graphitizing allomelanin, by laser or thermal treatment needs detailed investigation [70].

Effect of light in the synthesis and modification of allomelanin. Effect of light on allomelanin is still widely unexplored [71]. The possibility of using light at different wavelengths for allomelanin production or photochemical modification should be considered.

We believe that the issues listed above are very important in view of a broader development of the use of allomelanin for the design of new nanodevices.

## 6. Conclusions

This review has provided a comprehensive summary of the characterization, and natural occurrence and emerging applications of PDHN nanoparticles, primarily those synthesized from 1,8-DHN.

Our discussion highlighted that PDHN are typically monodisperse nanospheres, characterized by a broad UV-Vis absorption spectrum extending into the near-infrared region, a negative zeta potential indicating high colloidal stability, and an intrinsic porous structure confirmed by advanced electron microscopy. Molecular and chemical characterization, utilizing techniques, such as FTIR, XPS, and ESI-MS, consistently reveals the presence of quinone and phenolic functional groups, which are key to the material’s remarkable free-radical scavenging and antioxidant properties. These properties are further enhanced upon light exposure and are comparable to those of established antioxidants like ascorbic acid.

The unique combination of photothermal efficiency, redox activity, and biocompatibility has positioned PDHN NPs as promising candidates in diverse application fields, particularly in human health and materials science. PDHN NPs have been successfully employed in synergistic therapies, such as combining PTT with immunotherapy, utilizing their PTT efficiency and cargo-delivery capability. Furthermore, their rapid ROS scavenging ability is crucial for mitigating PTT-induced inflammation and enhancing diabetic wound healing when incorporated into hydrogel dressings. PDHN NPs have also shown potential for mitigating radiation-induced injury and providing protection against sensorineural hearing loss (SNHL). PDHN NPs also proved to be valuable for sustainable water remediation, acting as high-efficiency adsorbents for organic pollutants like dyes. They have also been incorporated into composite materials to enhance mechanical properties and provide wide-spectrum multi-radiation resistance, leveraging their broad-spectrum absorption. Their application extends to advanced separation, where a porous PDHN coating on MOFs allows for the selective separation of hexane isomers without compromising core porosity.

Overall, these findings demonstrate that PDHN-based nanomaterials, supported by their intrinsic optical absorbance, structural tunability, and potent radical-scavenging capacity, form a robust, multifunctional platform for diverse applications. In conclusion, PDHN NPs represent a highly versatile class of bio-inspired materials whose antioxidant and photothermal capabilities are already driving advances in biomedicine and materials science. Continued development of tailored PDHN NPs morphologies and compositions will further expand their impact across these emerging technological domains.

## Figures and Tables

**Table 1:** Schematic representation of text content of paragraph 3.

Application	Case Study	Specific Application	Structure	Precursor
Human Health	[46]	Treatment of Glioblastoma (GBM)	Biomimetic nanoplatform PDHN@CLP@CCM	1,8-DHN
	[47]	Inflammation prevention	DNA-guided PDHN nanodisks	1,8-DHN
	[48]	Diabetic wound healing	HA hydrogel dressing loaded with PDHN and BNN6	1,8-DHN
	[49]	Diabetic wound healing	Injectable hydrogel dressing (PQCS/OD/PDHN@Cur)	1,8-DHN
	[50]	Mitigate radiation-induced injury	Melanized bacteria	Natural Allomelanin
	[51]	Sensorineural hearing loss	Synthetically engineered allomelanin nanoparticles	1,8-DHN
	[52]	Detection of dopamine in human serum.	Novel composite (AM@UIO-66-NH2)	1,8-DHN
Material development	[53]	Wastewater treatment	Nylon membrane coated with PDHN	1,8-DHN
	[54]	Wastewater treatment	Ag/SE and Ag/PDHN NPs composites	Sepia eumelanin and 1,8-DHN
	[55]	Hexane isomers separation	PDHN coated Zr-MOF	1,8-DHN
	[28]	UV shielding film	PDHN NPs/PVA film	1,8-DHN
	[56]	UV shielding film	PDHN NPs/FPI film	1,8-DHN
	[57]	UV and ionizing radiation shielding film	PDHN/PU film	1,7-DHN and 2,3-DHN

**Table 2:** Schematic representation of text content of paragraph 4.

**Allomelanin in** **Nature**	**Case Study**	**Specific Application**	**Source**
	[58]	Allomelanin extraction	black knot fungus (*Apiosporina morbosa*)
	[59]	Effects of blue light on cap pigmentation	edible mushroom (*Morchella sextelata*)
	[60]	synthesis of PDHN	Streptomyces glaucescens and recombinant *Escherichia coli* BL21(DE3) strains

## Data Availability

No new data were created or analyzed in this study. Data sharing is not applicable to this article.
